# An Exceptional Case of Diabetic Ketoacidosis

**DOI:** 10.1155/2017/4351620

**Published:** 2017-03-26

**Authors:** Celine Van de Vyver, Jorn Damen, Carl Haentjens, Dominique Ballaux, Benoit Bouts

**Affiliations:** ^1^Ghent University, Gent, Belgium; ^2^Emergency Department, AZ Nikolaas, Sint-Niklaas, Belgium; ^3^Department of Endocrinology, AZ Nikolaas, Sint-Niklaas, Belgium

## Abstract

We present a case of diabetic ketoacidosis, known as one of the most serious metabolic complications of diabetes. We were confronted with rapid neurological deterioration and unseen glycaemic values, which reached almost 110 mmol/L, subsequently resulting in hyperkalaemia and life-threatening dysrhythmias. This is the first reported live case with such high values of blood glucose and a favourable outcome.

## 1. Introduction

Diabetic ketoacidosis (DKA) is known as one of the most serious complications of diabetes, besides hyperosmolar hyperglycaemic syndrome (HHS), and it is associated with significant morbidity and mortality. The symptoms are often nonspecific and there are many diseases that mimic the presentation. The clinical course usually evolves within a short time frame (<24 h). DKA exists of a triad of uncontrolled hyperglycaemia, metabolic acidosis, and increased total body ketone concentration [1]. These three criteria are needed for diagnosis. The most common precipitating factors of DKA are infections and discontinuation of or inadequate insulin therapy. Mainstays of treatment are correction of hypovolemia and hyperglycaemia, rapid administration of insulin, and electrolyte management.

Glycaemic values in DKA normally do not exceed 33 mmol/L. In contrast, blood glucose in HHS is often higher [2, 3]. We present a case of severe diabetic ketoacidosis with glycaemic values of almost 110 mmol/L, leading to neurologic sequelae and requiring more aggressive treatment. A similar case report detailing this presentation with a favourable outcome does not exist.

## 2. Case Presentation

We present the case of a 33-year-old woman with a rapid deterioration of haemodynamic and neurological status. She is known with brittle diabetes mellitus type 1, treated with continuous subcutaneous insulin infusion and there is a history of gastroparesis. A report, given by relatives, stated that she went to a party 36 h earlier and complained of abdominal pain and vomiting afterwards. She presented to the ambulance team with a Glasgow Coma Scale score dropping from 13 to 3 in only 10 minutes. She had sinus tachycardia, absent peripheral pulses, blood pressure (BP) 99/52 mmHg, and temperature 307 Kelvin. Clinical examination revealed severe dehydration, respiratory distress with a respiratory rate of 40/min and bilateral diffuse rhonchi, PEARRL, minor neck stiffness, and no petechiae. She required urgent intubation. After intubation, her systolic BP decreased to 50 mmHg. Initial finger-stick found glucose to be above range of detection and ketonemia above 8 mmol/L. During transport to the hospital, a nonsustained ventricle tachycardia (NSVT) occurred. The patient was found to have a severe metabolic acidosis with a pH of 6.74, bicarbonate of 5.3 mmol/L, pO_2_ 50.2 mmHg, and pCO_2_ 39.9 mmHg. Further laboratory investigation showed a serum glucose of 106.8 mmol/L, a white blood cell count of 32.8 × 10^9^/L, C-reactive protein 789.54 nmol/L, lactate 4.6 mmol/L, sodium 113 mmol/L, bicarbonate 6 mmol/L, potassium 6.7 mmol/L, calcium 2.0 mmol/L, phosphate 3.53 mmol/L, serum osmolality of 378 mOsm/kg, troponin I 0.463 *μ*g/L, blood urea level of 47.8 mmol/L, and serum creatinine level 332.4 *μ*mol/L. Urine toxicological screen was positive only for paracetamol (210.5 *μ*mol/L) and not ethanolemia.

A presumptive diagnosis of diabetic ketoacidosis (DKA), sepsis, vasoplegia, and acute renal failure was made and the patient was treated with aggressive IV fluids (crystalloids), norepinephrine, 300 meq of sodium bicarbonate, calcium chloride, and 20 units of actrapid insulin IV bolus, along with an insulin drip (10 U/h).

Since the most common precipitating factor of DKA is infection, a computed tomography brain and abdominal scan, urine culture, and chest radiography were performed. All were negative for signs of infection. The chest radiography showed some mild pulmonary edema. Further evaluation included a lumbar puncture. Ceftriaxone and acyclovir were given empirically. Subsequently, the lumbar puncture came back negative.

Her condition progressively improved over the following days and she was successfully extubated. Renal function normalized over two days. Due to continuing abdominal pain, an abdominal laparoscopy was performed without any specific findings.

## 3. Discussion

There is no existing literature documenting such a case of high hyperglycaemic ketoacidotic coma and a favourable evolution. Glycaemia in DKA rarely exceeds 33 mmol/L. Predictors of mortality are comorbidities, severe acidemia at presentation (arterial blood pH < 7.0), development of coma, or fever [4].

DKA is the result of abnormal metabolism of carbohydrate, protein, fat, and derangement of fluid and electrolyte homeostasis. There is a decrease in the net effective action of circulating insulin. Counter-regulatory stress hormones such as glucagon (causing increased glycogenolysis), epinephrine, cortisol, and stress hormone are elevated. This combination leads to the increased production of nonesterified fatty acids (NEFA) and glycerol from breakdown of triglycerides in DKA. Glycerol is used as a substrate for gluconeogenesis and the great amount of NEFA results in the production of ketone bodies. The clearance of ketone bodies is impaired due to low insulin concentrations, increased glucocorticoids, and decreased peripheral glucose utilization. The buffer capacity of bicarbonate is limited and a metabolic acidosis can occur [1, 3, 5].

Hyperglycaemia may be mild in the presence of a good renal function. But as the disease process evolves, glucose-induced osmotic diuresis leads to volume depletion and secondary renal failure, thus impeding further glucose excretion [1, 3, 5].

Normally, in metabolic acidosis, we would expect a low pCO_2_ because of the increase in ventilation. The respiratory response to rapid changes in serum HCO3^−^ is relatively slow. It often takes 12 to 24 hours for the maximal respiratory response to develop. The ketoacids produced by diabetics are only synthesized in the liver and the protons must be transported across the blood brain barrier before respiratory stimulation can occur [6, 7]. A clear relationship is demonstrable between the fall in serum HCO3^−^ and pCO_2_. An equation has been designed for this linear relationship by Albert et al. [8]: expected pCO_2_ = 1.5 (measured HCO3^−^) + 8 ± 2. Other equations also exist, by Fulop [9], as pCO_2_ = 7.27 + 1.57 × (HCO3^−^). To use these formulas, one must be certain that the metabolic disorder exists for at least 12–24 hours [6, 7]. In our case, we would expect a pCO_2_ of 17 mmHg. However, the pCO_2_ was 39.9 mmHg. Thus, the respiratory compensation for metabolic acidosis is impaired. The combination of mixed metabolic and respiratory acidosis occurs under some clinical situations. One of the situations is cardiopulmonary arrest. Simultaneous failure of respiration and tissue perfusion results in CO_2_ retention and lactic acidosis, which further augments acid production. This is an acceptable explanation for the high pCO_2_ in our case. Our patient needed urgent intubation because of respiratory distress and a low Glasgow Coma Scale score of 3, and she presented with high serum lactate levels (>4 mmol/L). Another condition, in which respiratory acidosis can occur, is pulmonary edema. Depending upon the severity of fluid accumulation, respiratory alkalosis or acidosis may develop. Development of circulatory overload and hydrostatic pulmonary edema is attributed to the acute shift of a substantial volume of fluid from the intracellular into the extracellular compartment. This shift is an osmotic consequence of solute accumulation in the extracellular compartment during development of hyperglycaemia. The magnitude of this shift affects the severity of the ensuing circulatory overload and one of the main factors in this process is the degree of hyperglycaemia [4, 7].

A third condition that can explain the elevated pCO_2_ is nonhydrostatic pulmonary edema in diabetic ketoacidosis. Diabetes mellitus may affect the structure and function of the lungs. The most consistent functional changes include reduced lung volumes, reduced pulmonary elastic recoil, and reduced capillary lung volume. This can cause clinical lung dysfunction under stressful conditions [4, 7].

These three theories (mixed metabolic and respiratory acidosis caused by respiratory failure and secondary tissue hypoperfusion and lactic acidosis, pulmonary edema, and lung dysfunction caused by diabetes mellitus) can explain the high pCO_2_ in our case.

The most common precipitating factor of DKA is infection. Since leukocytosis above 25 × 10^9^/L requires further investigation [1], a lumbar puncture (LP) was performed. In our case, the occurrence of coma can be due to the high elevation of osmolality (>320 mosm/kg) and the acidosis, since studies have established, respectively, a positive linear relationship between osmolality and mental obtundation, and the fact that acidosis impairs utilization of glucose in brain cells [1, 2, 10].

Our patient also suffered from continuing abdominal pain. This condition can be explained by the correlation of abdominal pain and the severity of acidosis, a classic mimic of acute abdomen on the one hand and by the presence of gastroparesis on the other hand [2]. Gastrointestinal manifestations are not associated with the severity of hyperglycaemia or dehydration. The pathogenesis of acidosis associated abdominal pain can be a central neurogenic response to increased ketone bodies and acidosis. Diabetic gastroparesis may cause severe symptoms as nausea, vomiting, bloating, and abdominal pain. Hyper- and hypoglycaemia may result in, respectively, delayed or accelerated gastric emptying. Changes in gastric emptying can cause fluctuations in blood glucose level since a mismatch occurs between the arrival of carbohydrates in the small bowel and insulin levels. This situation creates a vicious circle for the patient. Further investigation of abdominal pain in DKA should be reserved for patients who suffer from persisting pain after resolution of ketoacidosis [11–15].

The goal of therapy in DKA is the improvement of circulatory volume and tissue perfusion, the correction of hyperglycaemia and electrolyte imbalance, and resolution of ketosis.

Initial fluid therapy is aimed at expanding interstitial and intravascular volume and reestablishing adequate renal perfusion. The initial fluid of choice is isotonic saline, at the rate of 15–20 mL/kg body weight per hour. The subsequent choice for fluid replacement depends on the state of hydration, serum electrolyte levels, and urinary output. The administration of insulin without fluid replacement in hypotensive patients could worsen circulatory collapse [1, 3, 5, 16].

In DKA with mental obtundation, intravenous regular insulin by continuous infusion is the treatment of choice. A bolus is not necessary if patients are given hourly insulin infusion at 0.14 U/kg body weight/hour. If blood glucose does not fall by 10% in the first hour, an intravenous bolus of 0.14 U/kg should be administered. The dose of continuous infusion should not be changed [1, 3, 5, 10, 16].

Mild to moderate hyperkalaemia is frequently seen at presentation. Causes of hyperkalaemia are acidosis which displaces potassium from the cell to the extracellular space, proteolysis, and insulin deficiency. The above-mentioned therapy of fluid therapy and insulin infusion can cause hypokalaemia. Potassium replacement is initiated after serum levels fall below the upper level of normal (5–5.2 mmol/L) [1, 3, 5, 16].

Use of bicarbonate remains contradictory [1, 3, 17–19]. Studies showed that bicarbonate therapy does not alter patient outcomes or increase the rate of pH correction. Rapid correction of acidemia by bicarbonate can induce hypokalaemia and rebound metabolic alkalosis and it has been linked to worsening of tissue hypoxia. Increase of mental obtundation can occur, possibly explained by the preferential movement across the blood brain barrier of CO_2_ compared with bicarbonate. The occurrence of cerebral oedema is mainly prevalent in children and young adults. However, it has to be considered that severe acidemia rarely occurs without the presence of comorbid conditions. These conditions can alter the response to bicarbonate. Therefore, each case must be assessed individually. Overall, it is suggested to use bicarbonate for serum pH below 6.9 and in the presence of concomitant cardiogenic shock and respiratory or renal failure [1, 3, 17–19]. All were present in our case. Severe acidosis may lead to adverse vascular effects and, in our patient, severe dysrhythmias and impaired repolarisations further suggested major cardiac instability. In mixed metabolic and respiratory acidosis, elevation of blood pH requires simultaneous therapy of respiratory and metabolic components of the acidosis. Arterial pCO_2_ will increase as bicarbonate is given prior to optimizing ventilation, while lactic acidosis continues [7].

Serum phosphate at presentation is typically normal or high due to shifts across the cell membrane. During insulin therapy, phosphate reenters the intracellular compartment leading to a fall in serum phosphate concentrations. Potential complications include respiratory and skeletal muscle weakness, haemolytic anemia, and poor cardiac performance. Studies have shown no benefit of phosphate replacement on clinical outcome. However, it can be indicated in patients with cardiac dysfunction, anemia, and respiratory depression [1, 3, 5, 16].

The high blood glucose in our patient possibly can be explained by volume depletion and secondary renal failure, thus impeding further glucose excretion, and the presence of diabetic gastroparesis. Gastric emptying is unpredictable and it makes blood glucose difficult to control. In addition, due to the gastroparesis, the patient often deals with abdominal pain and vomiting, causing a delayed recognition of DKA. Our patient is now on the pancreas transplant waiting list.
